# Autism Spectrum Disorder and BRIEF-P: A Review and Meta-Analysis

**DOI:** 10.3390/children11080978

**Published:** 2024-08-13

**Authors:** Esperanza Bausela-Herreras

**Affiliations:** Department of Health Sciences, Public University of Navarra, 31006 Pamplona, Spain; esperanza.bausela@unavarra.es

**Keywords:** autism spectrum disorder, BRIEF-P, flexibility, executive functions, inhibition, Miyake model, working memory

## Abstract

Background: This research can facilitate the development of early detection tools for ASD by identifying specific patterns of deficits in executive functioning, validating the use of the BRIEF-P as a detection tool, and complementing information obtained from other evaluation instruments (Autism Diagnostic Interview—ADI—and Autism Diagnostic Observation Schedule—ADOS). Aims: To gain knowledge of the application and usefulness of the BRIEF-P in the evaluation of executive functions (EFs) in people with ASD in the early years of the life cycle. Method: In order to systematically examine this hypothesis, a meta-analysis was conducted to identify the executive profile (strengths and weaknesses) of children with ASD. Out of a total of 161,773 potentially eligible published articles from different databases, 13 appropriate articles were revised and 4 articles were selected. Studies that were included evaluated samples involving individuals with ASD aged 2 to 8 years and were published in English or Spanish during the period of 2012–2022. Results: The executive profile obtained from the application of the BRIEF-P in individuals with ASD was analyzed. It was identified that children with ASD, compared to typically developing children, show significantly clinical scores on the flexibility, inhibition, and global executive functioning scales. The results support the hypothesis of an executive deficit, with flexibility and inhibition being diagnostic markers for early and prompt identification of autism. Conclusions and Discussion: The results confirm deficits in flexibility, although they are not conclusive. This may be due to aspects related to methodology, whereby the studies (i) include very large and heterogeneous age groups, (ii) do not discriminate based on the level of competence, and (iii) use instruments for evaluating executive functions that are not validated or adapted for people with ASD. Another reason is the lack of consensus in the very operational definition of the executive functions construct, with the studies focusing mainly on the cold dimension while ignoring the hot dimension. From the perspective of therapeutic and treatment implications, executive dysfunction can impact adaptive skills in daily life and consequently the person’s autonomy.

## 1. Introduction

Research on autism spectrum disorder (ASD) has advanced significantly in identifying and understanding the various clinical manifestations of this neurodevelopmental disorder. There is variability in clinical presentation in the early years of development that is not easy to identify early. Studies that have analyzed neuropsychological profiles, particularly executive profiles, show heterogeneity, making it necessary to have tools that are easy to use for individuals who live with the child in question without professional experience in neuropsychological evaluation.

### 1.1. Autism Spectrum Disorder (ASD)

ASD includes different manifestations of an individual’s level of functioning. It is characterized by qualitative alterations in reciprocal social interaction and social communication, and the presence of repetitive behaviors and restricted interests. Based on two dimensions, three levels are differentiated: social communication, restricted interests, and repetitive behavior.

ASD is a lifelong neurodevelopmental disorder characterized by poor social interactions, communication impairments, narrow interests, and repetitive behaviors, which hinder a child’s adaptation and independence. Children with ASD often experience [1] delayed language development and exhibit rigidity, repetitiveness, and poor nonverbal skills. These challenges can lead to emotional and problem behaviors, causing distress for parents and educators. Therefore, assessing and addressing these issues through interventions is crucial for recovery and inclusion, with professionals needing to tailor learning experiences to support independent life skills.

There are various review studies that have been carried out with individuals who have ASD, which include Pennington and Ozonoff [2], Kercood, Grskovic, Banda, and Begeske [3], Leung and Zakzanis [4], and Geurts, Van Den Bergh, and Ruzzano [5].

### 1.2. Executive Functions and ASD

Executive functioning (EF) can be likened to an “orchestra conductor” construct that controls, organizes, and directs cognitive activity, behavioral activity, and emotional response [6]. Executive functions are not exclusively “cold”, i.e., cognitive, because they also include processes that are “hot”, i.e., emotional and behavioral control [7].

The first researchers to define EF in individuals with ASD were Damasio and Maurer [8], who established that the executive deficits that those individuals presented were analogous with damage to the frontal lobe affecting individuals.

Based on this study, we can review other publications [9] that have focused on the study of executive deficits in people with ASD, as follows: (a) Russell [10], in her classic work “Autism as an executive function disorder”, considers that the neurobiology of autism has characteristics that are very similar to those of the neurobiology of executive functioning. That consideration has been defended and maintained by other authors [11]; (b) Hill [12] published “Executive dysfunction in autism”, in which a review of three dimensions was carried out: planning, mental flexibility, and inhibition. The information that was obtained from this review concludes by highlighting the importance of developing more in-depth research on the different dimensions that make up the executive functioning construct in people with ASD, as well as understanding the neuroanatomical correlates of the different dimensions throughout the life of an individual; (c) Etchepareborda [13] published the article “Executive functions and autism”. That author, as well as those previously mentioned, agree by highlighting tremendous cognitive rigidity as the most relevant characteristic of ASD. The ‘dysexecutive’ explanation attempts to integrate neurobiological, cognitive, and behavioral data. Thus, individuals with ASD perform poorly in tasks that those studies evaluate, namely inhibition, flexibility, and planning. Specifically, people with ASD manage to solve second-order mental tasks, but not executive function tests; (d) Martos and Paula [14] conducted the study “An approach to executive functions in autism spectrum disorder”. They reviewed the behavioral manifestations and the state of the research on executive functions in individuals with ASD, and its impact on the abilities of planning, mental and cognitive flexibility, generativity, response inhibition, mental abilities, and sense of activity. The results of this review point to the difficulty of considering the executive hypothesis in people with ASD, as there are not enough intervention programs with proven efficacy that minimize the effects of executive dysfunction in autism; (e) Talero et al. [15] published “Autism Spectrum Disorder and executive function”. This empirical study aimed to evaluate the performance of people with ASD in executive function tests. The results of this study show that children with ASD are susceptible to presenting significant alterations in tasks related to executive functions. This dysfunction correlates with the severity of autism and varies by age. Finally, those authors consider that this alteration is not a phenomenon that occurs exclusively in ASD. Executive functioning is an area that has also been shown to be impaired in children with ASD (for a review, see [16,17]).

Some of the symptoms presented by people with ASD can be explained by a failure in the efficiency of certain skills linked to the executive system, including the following: self-control of action and thought, planning, inhibition capacity, working memory, monitoring of action, inhibition of automatic responses, and cognitive flexibility. These alterations tend to be grouped under the name of dysexecutive syndrome [18], observed in individuals with a tendency to act impulsively, while ignoring relevant information for the activity they would wish to perform. Likewise, individuals show difficulties in finding alternative solutions when they do not achieve the desired objective, and, in some cases, they are incapable of realizing the mistakes they made or anticipating the consequences of their actions.

Table 1 reviews, non-systematically, various current studies that point to a deficit in the flexibility dimension [13,14,19,20,21,22,23,24] and working memory [22] in individuals with ASD.

Although there is no doubt about the presence of executive dysfunction in ASD, this theory suffers from the following problems [32]: (i) firstly, the presence of executive dysfunction is not specific to ASD, for there are many other disorders that share deficits in executive functioning: ADHD, Tourette’s syndrome (TS), schizophrenia, and Parkinson’s disease also present as executive function disorders [33]; (ii) secondly, this theory suffers from a fundamental problem, which is the over-extension of the term executive function in order to refer to a wide range of capacities that, in many cases, are not clearly defined. More importantly, not all of these capacities are affected in ASD [34].

We agree with Di Renzo et al. [35], who highlight both the difficulty of assessing executive functioning (a complex and heterogeneous construct) and ASD, while aiming at (i) a multisource research perspective and (ii) an approach that would allow a more accurate and complete assessment of executive functioning in ASD, while (iii) differentiating the clinical characteristics, and (iv) considering how the different components of development interact to determine their cognitive and social competence.

### 1.3. Behavior Rating Inventory of Executive Function—Preschool Version (BRIEF-P)

The Behavior Rating Inventory of Executive Function—Preschool Version (BRIEF-P) [36] is a questionnaire with which to assess the executive functioning of children (aged 2 years to 6 years), which is derived from the BRIEF [37] in the context itself and includes the dimension of self-regulation. In its development, Isquith et al. [36] relied on a series of models that converge on the idea that executive functions are a series of interrelated dimensions or processes, which are responsible for directing behavior and cognitive activity towards an objective.

Using a Likert-type frequency scale with three response options (never, sometimes, and frequently), parents and teachers (as informants) respond to the question: “How often have these behaviors been a problem compared with other children of the same age?” For this, it is necessary that the informants have known the child for a minimum period of 6 months.

The questionnaire is made up of 63 items under five clinical scales (inhibition, emotional control, flexibility, planning and organization, and working memory); three indices (inhibitory self-control, flexibility, and emerging metacognition); a global executive function index; and two validity scales (negativity and inconsistency). It takes 10–15 min.

It is an efficient and valid instrument with which to detect, assess, and monitor the development of executive functioning in children, and it is easy to use, brief, and structured.

So far, few studies have applied the BRIEF-P instrument (Behavior Rating Inventory of Executive Function—Preschool Version) to populations with ASD (see [38,39]). This is partly due to the complexity involved in the early and standardized diagnosis of ASD. The accurate and timely identification of this disorder is a significant challenge, as symptoms can vary widely and manifest differently in each individual. The limited availability of research in this area highlights the need to develop and validate specific and sensitive assessment tools to detect the particularities of executive functioning in young children with ASD.

### 1.4. Aim and Research Question

Research on ASD has advanced significantly in identifying and understanding the various clinical manifestations of this neurodevelopmental disorder. There is variability in clinical presentation in the early years of development that is not easy to identify early. Studies that have analyzed neuropsychological profiles, particularly executive profiles, show heterogeneity, making it necessary to have tools that are easy to apply by people who live with the child without professional experience in neuropsychological evaluation.

In this context, we review the findings published in relation to the application of the BRIEF-P in individuals with ASD who are of preschool age, in order to analyze the profile of executive functioning by answering the following question: Is it possible to obtain an executive functioning profile in children with ASD using the BRIEF-P?

This systematic review aims to answer the following question: How does cognitive age that permits the use of the BRIEF-P relate to the assessment of executive functions (flexibility, inhibition, and working memory) in individuals with ASD diagnosed using standardized tests, and what role do standardized instruments (hetero-report and/or self-report) play in these results?

This research can facilitate the development of early detection tools for ASD by identifying specific patterns of deficits in executive functioning, validating the use of the BRIEF-P as a detection tool, and complementing information obtained from other evaluation instruments (Autism Diagnostic Interview—ADI—and Autism Diagnostic Observation Schedule—ADOS). This has the potential to improve the accuracy and effectiveness of early detection and early interventions for children with ASD. Integrating findings from the BRIEF-P administered by parents and caregivers with data from tools such as ADI and ADOS administered by professionals can increase the reliability of early detection. The triangulation of data from multiple sources allows for better and earlier identification of children at risk of ASD.

## 2. Method

This systematic review of the scientific literature examines executive functioning in children with ASD, following the procedure established by the PRISMA statement for conducting systematic reviews [40] and examples such as [41].

### 2.1. Selection Process

**Figure 1:**
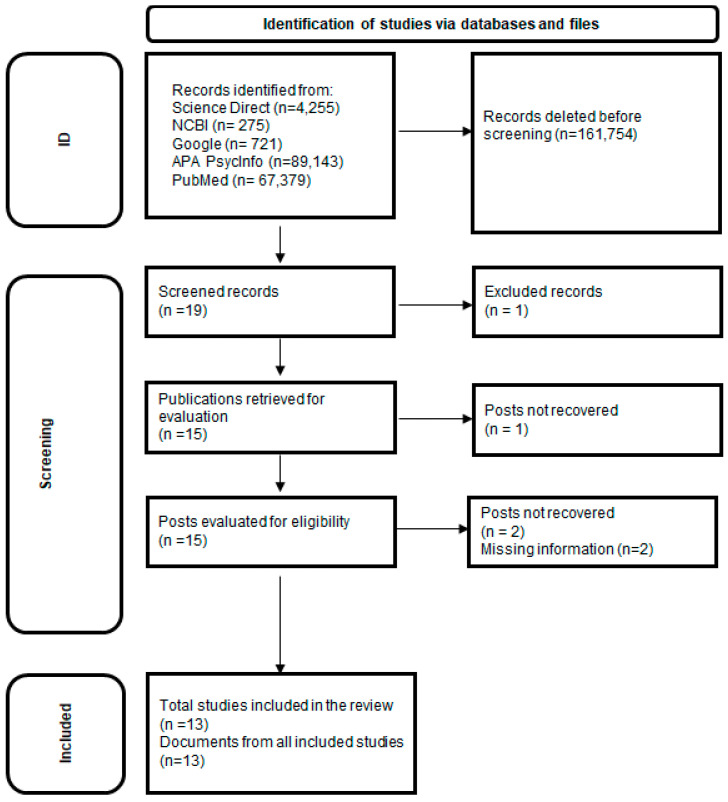
PRISMA flow diagram (based on the work of Page et al. [40]).

We have followed the guidelines of PRISMA 2020 [40] (see Figure 1), which were operationalized in a series of stages: (i) formulation of the problem; (ii) literature search; (iii) coding of the studies; (iv) statistical analysis and interpretation; and (v) publication of the results.

For the selection of keywords and, consequently, the studies, the PECO strategy (Participants, Interventions, Comparisons, Outcomes, Study design) by Morgan et al. [42] was used, with the following results: (i) Population: people with a diagnosis of autism spectrum disorder (ASD) obtained through the application of standardized tests (ADOS and or ADI); (ii) Exposure: cognitive age that allows for the application of the BRIEF-P; (iii) Comparison: executive dimensions, which may be a single construct or basic dimensions such as flexibility, inhibition, and working memory; (iv) Outcome: assessment of executive functions using standardized instruments (hetero-report and/or self-report) BRIEF-P.

### 2.2. Selection Criteria

The criteria were previously decided by considering the objectives of the study.

The *inclusion criteria* were as follows: (a) participants: people with a diagnosis of ASD obtained from the application of standardized tests; (b) cognitive age: that allows for the application of the BRIEF-P; (c) cognitive competence: obtained through the application of standardized tests; (d) executive dimensions: single construct or basic dimensions (flexibility, inhibition, and working memory); (e) assessment instruments: standardized to assess executive functions: hetero-report and/or self-report; (f) types of studies: empirical; (g) languages: English and Spanish; and (h) other features: full text.

The exclusion criteria were as follows: (a) participants: no diagnosis of ASD; (b) cognitive age: does not allow for the application of the BRIEF-P; (c) cognitive competence: not available; (d) assessment instruments: not standardized; (e) types of studies: case study and review; (f) language: other than English and Spanish; and (g) other characteristics: summary.

We carried out a systematic review and considered the following series of characteristics that define this type of scientific publication: (i) the preparation process is specified in detail; (ii) it is possible to replicate and verify the results and conclusions; (iii) an issue is dealt with in depth; (iv) the research is useful for obtaining concrete answers to specific clinical questions; and (v) the research provides objective data.

### 2.3. Databases Searched

We conducted a literature search in Science Direct, NCBI (National Center for Biotechnology Information), Science Direct, APA PsycInfo, and PubMed.

### 2.4. Search Strategy

The keywords used in the search were as follows: autism or ASD or ASD; and Behavior Rating Instrument of Executive Function-Preschool or BRIEF-P.

The search language was primarily English. The search itineraries focused on the last ten years. We obtained the following results:

(i) Science Direct → 4255 results:
  Search 1 → 97 results
  (autism or ASD) and (Behavior Rating Instrument of Executive Function-Preschool);
  Search 2 → 4.158 results
  (autism or ASD) and (BRIEF-P).
(ii) NCBI → 275 results:
  Search → NCBI → Total 142
  (autism+or+ASD) and (BRIEF-P);
  Search → NCBI → Total 133
  ((“autistic disorder”[MeSH Terms] OR (“autistic” [All Fields] AND “disorder”[All Fields]) OR “autistic disorder”[All Fields] OR “autism”[All Fields]) OR “asd”[All Fields]) AND BRIEF-P[All Fields] AND (“2012/02/07”[PDat] : “2022/02/03”[PDat]) (autism+or+ASD) and (BRIEF-P).
(iii) APA PsycInfo → 89,143 results:
  Search → autism or ASD and BRIEF-P → 89,143 results.
(iv) PubMed:
  Search → autism or ASD and BRIEF-P → 67,379 results.
  The search strategy is reflected in the PRISMA flow diagram (Figure 1).

In addition to adapting the syntax to the requirements of each database, specific filters for each database were applied in order to facilitate the search according to the established inclusion and exclusion criteria.

### 2.5. Risk of Bias

The methodological quality of the selected studies was assessed using the QUADAS-2 tool (Whiting et al., 2003) [43] (see Table 2).

## 3. Results

The search returned 161,773 potentially eligible studies. Finally, 13 works that had been published in the last ten years were selected, because they met the necessary criteria to be part of the present study. The selected articles were reviewed, and the most relevant information was extracted.

A total of 13 studies were ultimately selected, forming the basis of the present systematic review. Initially, 161,754 records were obtained. In the first screening process, those that, based on the information provided by the title, were unrelated to the study’s objective and did not meet one or more inclusion criteria (empirical study, participants’ age, standardized assessment of cognitive and/or executive competence) were excluded. Subsequently, through the reading of abstracts, a second screening and suitability assessment was conducted, ultimately selecting 19 full texts, of which 13 met the established requirements. The flowchart of the study selection process can be seen in Figure 1.

### 3.1. Reviewed Studies

Table 3 includes the reviewed studies. Table 3 was created to record the information obtained in a structured manner as follows: Author, Year, Country, Title, Design, Sample Size, Median Age of Sample, Diagnostic Instrument for ASD, Assessment Instrument for EF, Assessment Instrument for Other Competencies, and Executive Profile.

### 3.2. Meta-Analysis

We have selected four studies that have sufficient statistical information to perform the meta-analysis with six comparisons (ADS versus control) that allow us to compare the results in relation to the scores on the clinical inhibition and flexibility and global executive functioning scales.

The Q test for homogeneity indicates that we cannot reject the null hypothesis that the effect size is the same in all studies.

The study that has the most weight in the meta-analysis is the one developed by Jahromi et al. [54].

In this case, the overall effect measures the statistical significance of the result of the meta-analysis. In our study, it was observed that it is statistically significant as the value is less than 0.05 (for 95% CI). There are statistically significant differences in BRIEF-P scores between people with ASD and the control group.

Considering a significance level at 0.05, a *p*-value of 0.015 would be less than this threshold and would be considered statistically significant.

**Figure 2:**
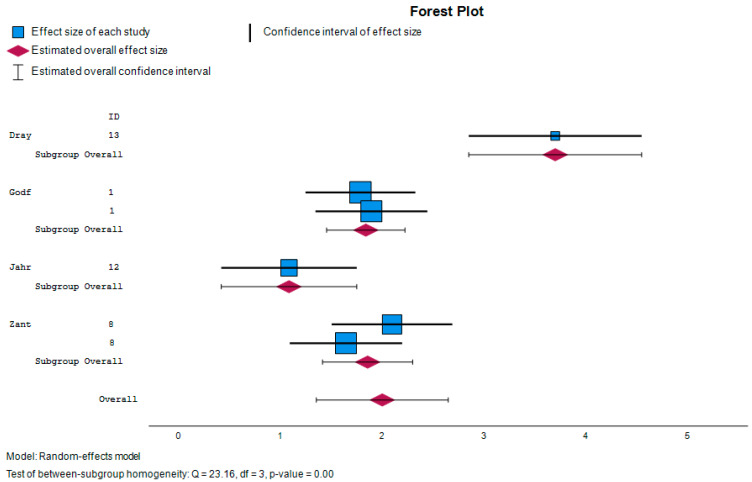
Forest plot [44,51,54,55].

Figure 2 presents the forest plot, which visually summarizes the results of the meta-analysis. Each study is represented by a horizontal line indicating its confidence interval, with a square marker denoting the effect size. The overall effect estimate is illustrated by a diamond at the bottom of the plot, providing a comprehensive view of the combined data. This visualization helps to quickly assess the consistency and significance of the findings across different studies.

## 4. Discussion and Conclusions

We start from our research question: How is the cognitive age that permits the use of the BRIEF-P related to the assessment of executive functions (flexibility, inhibition, working memory) in individuals with ASD diagnosed through standardized tests, and what role do standardized instruments (hetero-reports and/or self-reports) play in these outcomes?

The summary of the presented studies provides a comprehensive view of executive function (EF) deficits related to autism spectrum disorder (ASD) and other neuropsychiatric disorders. Pennington and Ozonoff [2] conducted a pioneering review of studies on ASD, ADHD, conduct disorder (without ADHD), and Tourette’s syndrome. They found that EF deficits are common in both ADHD and autism, but not in conduct disorder or Tourette’s. Furthermore, the severity and profile of these deficits differ between ADHD and autism, being more severe in autism. Kercood et al. [3] indicated that individuals with ASD score lower in working memory, cognitive flexibility, and planning compared to individuals with neurotypical development. Additionally, a low verbal working memory score is associated with greater adaptive behavior problems and more restrictive and repetitive behaviors. Leung and Zakzanis [4] found that deficiencies in this area do not uniformly characterize all individuals with ASD. Geurts et al. [5] found that individuals with ASD show poorer performance in tasks of automatic response inhibition and interference control, although they noted that variables such as age and IQ may influence these results. Ratto et al. [48] showed fewer EF problems and repetitive behaviors, suggesting a possible positive “bilingual effect” on EF in neurodevelopmental disorders. Otterman et al. [56] suggested a gradual association of EF difficulties along the ASD and ADHD continuum, indicating that EF problems may be precursors to traits of these disorders from an early age. Stephens et al. [49] found a relationship between childhood behaviors indicative of ASD risk and EF in early childhood, underscoring the importance of these behaviors as early indicators. Finally, Di Renzo et al. [35] concluded that the use of the BRIEF-P instrument, completed by parents, provides greater knowledge of a child’s EF, although it does not add additional clinical data to the diagnosis.

Together, these studies highlight the complexity of EF deficits associated with ASD and other disorders, emphasizing the need to consider multiple factors and contexts when evaluating these difficulties.

We can conclude that studying executive functions such as flexibility, inhibition, and working memory is crucial for understanding cognitive development in individuals with autism spectrum disorder (ASD). These functions are fundamental for self-regulation and adaptive behavior and are often impaired in individuals with ASD. The use of instruments like the BRIEF-P (Behavior Rating Inventory of Executive Function—Preschool Version) has been implemented to assess these functions in children, but it is essential to understand how cognitive age influences the applicability and accuracy of this tool in populations with ASD. Additionally, the reliability and validity of the results can be affected by the type of instrument used, whether it be hetero-reports (evaluations conducted by third parties, such as parents or teachers) or self-reports (self-assessment by the individual).

The results of our research indicate that cognitive age is a determining factor for the effective use of the BRIEF-P in assessing executive functions in individuals with ASD. It is observed that, as cognitive age approaches the normative developmental stage for the use of the BRIEF-P, the accuracy of assessing executive functions such as flexibility, inhibition, and working memory improves significantly. This suggests that assessment tools need to be carefully adapted to the individual’s cognitive level to obtain a valid and reliable evaluation.

Furthermore, standardized assessment instruments play a crucial role in obtaining accurate results. Our study highlights that the use of both hetero-reports and self-reports can offer complementary perspectives, although discrepancies may also occur due to differences in perception and introspection. Therefore, it is advisable to use a combination of both types of reports to gain a more comprehensive and accurate view of executive functioning in individuals with ASD.

Executive functioning is an affected area in children with ASD (for a review, see Best and Miller [16]). Kenworthy et al. [17], in comparing children with and without ASD, found significant improvements in parent-reported general executive functioning after parent–child interaction therapy (PCIT). The changes that were experienced before and after therapy in emotional regulation scores were related to executive functioning.

In conclusion, the appropriate selection and adaptation of assessment tools, considering cognitive age and the type of report, is essential for an accurate assessment of executive functions in individuals with ASD. This not only enhances the understanding of their capabilities and limitations but also facilitates the development of more effective and personalized interventions. These results are in line with those obtained by various researchers who have applied the BRIEF-P to children with ASD, as follows: (i) McClain et al. [57], in comparing children with ASD versus children with intellectual disabilities, found that the latter exhibited more significant deficiencies in EF, regardless of diagnosis, with working memory being the most affected executive function ability in both groups; and (ii) Godfrey et al. [44] confirmed a greater difficulty in switching attention between activities and persistence in interests in individuals with ASD.

Based on the results obtained, we suggest including the BRIEF-P in the protocol for evaluating the executive functions of children with ASD, making it necessary to complete it using information obtained from the informants (parents, caregivers, and teachers) by adding the information gained from the child him/herself, thus evaluating the three nuclear dimensions of executive functioning proposed by [58] as follows: working memory, inhibition, and flexibility.

The results are not conclusive. This may be due to various aspects that are related to methodology. For example, a large proportion of the studies have been carried out with children and adolescents of different ages and IQs below the normal range. Studies with adult samples have used individuals with IQs within the normal range or above. Another reason is that, in comparative studies, the control groups used are not always the most appropriate for comparison with individuals with ASD.

### 4.1. Practical Implications of the Study

The research highlights several practical implications of using the BRIEF-P tool for children with autism spectrum disorder (ASD), as follows: (i) improved screening and diagnosis: the BRIEF-P helps accurately identify executive function (EF) impairments, differentiating them from other developmental disorders; (ii) targeted interventions: identifying specific EF weaknesses enables the creation of personalized intervention strategies, enhancing therapeutic and educational outcomes; (iii) informed educational strategies: insights from BRIEF-P assessments guide educators in tailoring classroom strategies and supports for students with ASD; (iv) parental and caregiver guidance: the tool provides valuable information for parents to support their child’s development at home, improving communication with professionals; (v) research and policy development: the findings inform policy regarding early screening and intervention, advocating for the inclusion of EF assessments in standard diagnostic procedures; (vi) longitudinal tracking and outcome prediction: the regular use of the BRIEF-P enables the tracking of EF development over time, helping predict long-term outcomes and adjust interventions; and (vii) cross-setting consistency: evaluating EF across multiple settings ensures comprehensive support for children with ASD. In summary, the study underscores the importance of a comprehensive, individualized, and context-sensitive approach to supporting the development of executive functioning skills in children with ASD.

### 4.2. Study Limitations and Future Outlook

The BRIEF-P is valuable for outlining developmental profiles in preschoolers but is insufficient for gauging the severity of autistic symptoms. For children with ASD, it can help define executive functioning as a “specifier”, per DSM-5-TR guidelines. However, an executive function assessment should be complemented by a clinical diagnosis from a multidisciplinary team with expertise in autism.

We mention the following study limitations: (i) differences in the presentation of executive functions: It is important to consider individual differences in the presentation of executive functions. These include the following: (a) intra-individual variability: children with ASD may exhibit inconsistent performance in executive function tasks due to factors such as stress, fatigue, or contextual demands; (b) gender differences: research has suggested that there are differences in the manifestation of executive function difficulties between boys and girls with ASD; and (ii) influence of contextual and environmental factors: it is relevant to examine how the environment (family, school, social) influences the development of executive functions.

Next, we analyze the following questions [12] that can guide future studies: (i) What is the influence of IQ on executive performance in people with ASD?; (ii) What neural mechanisms correspond to specific executive functions in individuals with and without ASD?; (iii) What is the profile of executive function and executive dysfunction in people with ASD throughout their lives?; (iv) In individuals with ASD who do not show clear executive deficits in laboratory tasks, how do they perform in naturalistic settings that involve executive functions, such as shopping, for example?; (v) Is there a specific profile of executive dysfunction that is exclusive to people with ASD, and different from other neurodevelopmental disorders that are associated with executive dysfunction?; and finally (vi) What would a cognitive model of executive functions in individuals with ASD look like?

## Figures and Tables

**Table 1:** Executive deficits in people with ASD (own elaboration).

**Study**	**Title**	**Neurodevelopmental Disorder**	**Executive Deficits**
Blijd-Hoogewys et al. [19]	Executive Functioning in Children with ASD: An Analysis of the BRIEF	ASD	Flexibility
Brady et al. [20]	Conceptual and Perceptual Set-shifting executive abilities in young adults with Asperger’s syndrome	ASD (syndrome de Asperger)	Conceptual flexibility
Campbell et al. [21]	Nonverbal, rather than verbal, functioning may predict cognitive flexibility among persons with autism spectrum disorder: A preliminary study	ASD (no verbal)	Flexibility
Chen et al. [22]	Deficits in executive functions among youths with autism spectrum disorders: An age-stratified analysis	ASD	Working memory and planning in youth (13–18 years) Flexibility in children (8–12 years old)
Etchepareborda [13]	Funciones ejecutivas y autismo	ASD	Inhibition, flexibility, and planning
Geurts et al. [5]	Prepotent response inhibition and interference control in autism spectrum disorders: Two Meta-Analyses	ASD	Inhibition, working memory, cognitive flexibility, and planning
Hüpen et al. [25]	Performance monitoring in autism spectrum disorders: A systematic literature review of event-related potential studies	ASD	Self-monitoring of learning
Kloosterman et al. [26]	Executive functioning as a predictor of peer victimization in adolescents with and without an Autism Spectrum Disorder		Emotional control
Landry and Al-Taie [23]	A Meta-analysis of the Wisconsin Card Sort Task in Autism	ASD	Rigidity (not flexibility)
Martos-Pérez and Paula-Pérez [27]	Una aproximación a las funciones ejecutivas en el trastorno del espectro autista	ASD	Planning, mental and cognitive flexibility, and response inhibition
Pellicano et al. [28]	Executive function predicts school readiness in autistic and typical preschool children	ASD	Inhibition and working memory
Talero et al. [15]	Trastorno del espectro autista y función ejecutiva	ASD	Executive function (unitary construct)
Van Eylen et al. [24]	Executive functioning and local-global visual processing: candidate endophenotypes for autism spectrum disorder?	ASD	Flexibility, fluency, and inhibition of automatic responses
Vanegas and Davidson [29]	Investigating distinct and related contributions of Weak Central Coherence, Executive Dysfunction, and Systemizing theories to the cognitive profiles of children with Autism Spectrum Disorders and typically developing children	ASD	Executive function (unitary construct)
Wu et al. [30]	Executive function in high-functioning autism: Decision-making consistency as a characteristic gambling behaviour	ASD	Repetition and rigidity
Yi et al. [31]	Event-based prospective memory in children with autism spectrum disorder: The role of executive function	ASD	Prospective report

Note: Sorted alphabetically.

**Table 2:** Effect size estimates.

Author/Country	Probability of Biases				Concern about the Applicability of the Results		
	Selection of Individuals	Index Test	Reference Test	Flow and Timing	Selection of Individuals	Index Test	Reference Test
Godfrey et al. [44]/Canada	high	low	low	uncertain	low	low	low
Parladé et al. [45]/Canada	low	low	uncertain	uncertain	low	uncertain	uncertain
Nuske et al. [46])/EE.UU	high	uncertain	uncertain	uncertain	high	low	low
Kitzerow et al. [47]/Germany	high	low	low	low	high	low	low
Ratto et al. [48]/EE.UU	high	low	low	high	low	low	low
Stephens et al. [49]/EE.UU	low	low	low	uncertain	low	low	low
MacFarlane et al. [50]/EE.UU	high	low	low	uncertain	high	low	low
Zantinge et al. [51]/The Netherlands	high	low	low	uncertain	high	low	low
Gorman et al. [52]/EE.UU	high	low	low	uncertain	low	low	low
Di Renzo et al. [35]/Italy	high	high	low	uncertain	high	low	low
Smithson et al. [53]/EE.UU	high	high	low	uncertain	high	low	low
Jahromi et al. [54]/EE.UU	high	high	uncertain	uncertain	low	uncertain	high
Drayer [55]/EE.UU	high	high	low	uncertain	high	low	low

**Table 3:** Studies included in the review (own elaboration).

Author/Country	Title	Design	Sample/n	Sample/Median Age	Diagnostic Instrument_ASD	Assessment Instrument for EF	Assessment Instrument for Other Competencies	Executive Profile	Included in the Meta-Analysis
Godfrey et al. [44]/Canada	Autism interest intensity in early childhood is associated with executive functioning but not reward sensitivity or anxiety symptoms.	Non-experimental; Descriptive; Comparative/causal; Two groups: - ASD; - Comparison.	- ASD = 33; - Comparison = 42.	3–6 years	> Social Responsiveness Scale–Second Edition (SRS-2)	BRIEF-P	> Interests Scale (IS); > Behavioral Inhibition and Behavioral Approach System–Parent Version (BISBAS); > Behavior Assessment System for Children–Third Edition (BASC-3).	> The group with ASD had greater difficulties on the flexibility clinical scale and on the inhibitory control index.	YES
Parladé et al. [45]/Canada	Parent–child interaction therapy for children with autism spectrum disorder and a matched case–control sample.	Empirical; Quasi-experimental; Measures: pre-test and post-test; Two groups: - ASD; - Control.	- ASD = 16; - Comparison = 16.	3–7 years	> The Autism Diagnostic Observation Schedule, 2nd-edition (ADOS-2).	BRIEF-P	> Differential Abilities Scale, Second Edition (DAS-II); > Picture Vocabulary Test, Fourth Edition (PPVT-4); > Expressive Vocabulary Test, Second Edition (EVT-2); > Peabody; > Parenting Stress Index, Fourth Edition: Short Form (PSI-4: SF); > ECBI; > Behavior Assessment System for Children, Second Edition, Parent Rating Scale (BASC-2 PRS); > Social Responsiveness Scale, Second Edition (SRS-2).	> There are no statistically significant differences in the BRIEF-P global executive functioning scale between pre- and post-treatment.	NO
Nuske et al. [46]/EE.UU	Self-Regulation is Bi-Directionally Associated with Cognitive Development in Children with Autism.	Non-experimental; Descriptive; Comparative/causal; Two groups: - Minimally verbal; - Typically verbal.	ASD: - Minimally verbal children = 38; - Typically verbal children = 46.	8 years	> Differential Ability Scales-II (DAS-II)	BRIEF-P	> Behavioral Interference Coding Scheme (BICS)	> Children with ASD are at high risk of self-regulation difficulties. > Children with ASD compared with typically verbal had more self-regulation difficulties.	NO
Kitzerow et al. [47]/Germany	Study protocol of the multi-centre, randomised controlled trial of the Frankfurt Early Intervention Programme A-FFIP versus early intervention as usual for toddlers and preschool children with Autism Spectrum Disorder (A-FFIP study).	Quasi-experimental; One group with six measurements.	ASD = 134	24–66 months (2–5.5 years)	> Autism Diagnostic Interview—Revised (ADI-R); > Autism Diagnostic Observation Schedule, 2nd-edition (ADOS-2).	BRIEF-P	> Brief Observation of Social Communication Change (BOSCC); > Social Responsiveness Scale—short version (SRS-16); > Repetitive Behavior Scale—Revised (RBS-R); > Child Behavior Checklist 1 ½–5 (CBCL 1 ½–5); > Parent sense of competence scale (PSOC); > Depression Anxiety and Stress Scale—short form (DASS-21); > Family Quality of Life Survey (FQOLS); > Early Social Communication Scale (ESCS); > Dyadic Communication Measure for Autism (DCMA); > Bayley Scales of Infant and Toddler Development 3rd Edition (Bayley-III); > Parent Adherence to Treatment and Competence Scale (PATCS)	> In preschool children with ASD, EF disturbances were observed in the real world that were not related to ASD symptoms.	NO
Ratto et al. [48]/EE.UU	Parents report fewer executive functioning problems and repetitive behaviors in young dual-language speakers with autism.	Non-experimental; Descriptive. Comparative/causal; Two groups: - Bilingual; - Monolingual.	ASD: - Bilingual = 24; - Monolingual = 31.	Bilingual = 4.73 (0.57); Monolingual = 4.76 (0.67).	> The Autism Diagnostic Observation Schedule, 2nd-edition (ADOS-2)	BRIEF-P	> Social Responsiveness Scale-2 (SRS-2); > Vineland-II.	> The bilingual advantage of EF observed in children with normotypical development may also be extended to young children with ASD.	NO
Stephens et al. [49]/EE.UU	Infant quantitative risk for autism spectrum disorder predicts executive function in early childhood	Non-experimental;.	ASD = 585	42 months (3.5 years)	> The First Year Inventory 2.0 (FYI 2.0)	BRIEF-P	> Social Responsiveness Scale, Second Edition (SRS-2.0)	> Certain childhood behaviors related to ASD are linked to EF difficulties in early childhood	
MacFarlane et al. [50]/EE.UU	Quantitative analysis of disfluency in children with autism spectrum disorder or language impairment	Non-experimental; Descriptive; Comparative/causal; Three groups: - ASD; - Language impairment; - Control.	- ASD = 47; - Control = 32; - Language impairment = 18.	4–8 years	> Autism Diagnostic Observation Schedule (ADOS)	BRIEF-P	> Verbal IQ (VIQ), performance IQ (PIQ), and full-scale IQ (FSIQ) using the Wechsler scales tests; > CELF Preschool-2; > Communication Checklist (CCC-2).	> Executive functioning difficulties are more common in individuals with ASD than in children with language disorders.	NO
Zantinge et al. [51]/The Netherlands	Physiological Arousal and Emotion Regulation Strategies in Young	Non-experimental; Descriptive; Comparative/causal; Two groups: - ASD; - Control.	- ASD = 27; - Control = 44.	41–81 months (3.4–6.75 years)	> Autism Diagnostic Interview-Revised (ADI)	BRIEF-P	> Dutch Wechsler Nonverbal Scale of Ability (WNV-NL); > Wechsler Preschool and Primary Scale of Intelligence (WPPSI-III-NL); > Nonverbal Intelligence Test (SON-R 2.5–7); > Mullen Scales of Early Learning (MSEL); > Wechsler Nonverbal Scale of Ability (WNV); > Social Skills Rating System (SSRS); > Peabody Picture Vocabulary Test-III-NL (PPVT-III-NL); > Locked Box Task.	> Children with ASD had significantly more problems with inhibitory control and mental flexibility compared to children in the control group.	YES
Gorman et al. [52]/EE.UU	Uh and um in Children With Autism Spectrum Disorders or Language Impairment	Non-experimental; Descriptive; Comparative/causal; Three groups: - ASD; - Control; - Specific language impairment.	- ASD = 50; - TD = 47; Specific Language Impairment = 17.	4–8 years	> Autism Diagnostic Observation Schedule (ADOS)	BRIEF-P	> Children’s Communication Checklist (CCC-2); > Social Communication Questionnaire.	> There were no reliable associations between the um–:uh ratio and chronological age, intelligence, or executive function.	
Di Renzo et al. [35]/Italy	Assessment of Executive Functions in Preschool-Aged Children with Autism Spectrum Disorders: Usefulness and Limitation of BRIEF-P in Clinical Practice	Non-experimental; Descriptive; Comparative/causal; Four groups: - ASD; - Autistic children; - Autism spectrum children; - Children at risk of autism.	- ASD = 46; - AUT = 26; - SpD = 7; - Risk = 13.	24–76 months (2–6.3 years)	> The Autism Diagnostic Observation Schedule, 2nd-edition (ADOS-2)	BRIEF-P	> Leiter International Performance Scale-Revised (Leiter-R)	> BRIEF-P is not indicative of the severity of autistic symptoms.	NO
Smithson et al. [53]/EE.UU	Real World Executive Control Impairments in Preschoolers with Autism Spectrum Disorders	Non-experimental; Descriptive; Comparative/causal; Two groups: - ASD; - Control.	- ASD = 39; - Control = 39.	2.83–5.83 years	> Autism Diagnostic Observation Schedule (ADOS)	BRIEF-P	> Wechsler Preschool and Primary Scale of Intelligence-Revised (WPPSI-R); > Wechsler Preschool and Primary Scale of Intelligence-Third Edition (WPPSI-III);	> Preschoolers with ASD showed generalized deficits in EF control when informants were parents.	NO
Jahromi et al. [54]/EE.UU	The importance of self-regulation for the school and peer engagement of children with high-functioning autism	Non-experimental; Descriptive; Comparative/causal; groups: - ASD; - Control.	- ASD_high performance = 20; - Control = 20.	54.57 months (4.55 years)	> Autism Diagnostic Interview-Revised (ADI-R)	BRIEF-P	> Child Behavior Questionnaire–Short Form (CBQ-SF)	> Emotion regulation was positively related to performance in the Day/Night inhibition task, and was negatively related to deficits on the inhibitory control index in the BRIEF-P.	YES
Drayer [55]/EE.UU	Profiles of executive functioning in preschoolers with autism	Non-experimental; Descriptive; Comparative/causal; Two groups: - ASD; - Pervasive developmental disorder.	- ASD = 29; - Control = 30.	6 years	Schooled in a Special Education center being consulted on the clinical diagnosis.	BRIEF-P	> Leiter International Performance Scale-Revised (Leiter-R)	> ASD reported a higher level of difficulty in EF in all domains, compared to the group of children without autism.	YES

Note: Sorted chronologically.
